# MTR3D‐AF2: Expanding the coverage of spatially derived missense tolerance scores across the human proteome using AlphaFold2

**DOI:** 10.1002/pro.5112

**Published:** 2024-07-19

**Authors:** Aaron S. Kovacs, Stephanie Portelli, Michael Silk, Carlos H. M. Rodrigues, David B. Ascher

**Affiliations:** ^1^ The Australian Center for Ecogenomics, School of Chemistry and Molecular Biosciences The University of Queensland Brisbane Queensland Australia; ^2^ Computational Biology and Clinical Informatics Baker Heart and Diabetes Institute Melbourne Australia; ^3^ Centre for Population Genomics, Murdoch Children's Research Institute Melbourne Australia; ^4^ Systems and Computational Biology Bio21 Institute, The University of Melbourne Melbourne Australia

**Keywords:** missense mutation, pathogenicity prediction, population variation, protein structure prediction, selective pressure

## Abstract

The missense tolerance ratio (MTR) was developed as a novel approach to assess the deleteriousness of variants. Its three‐dimensional successor, MTR3D, was demonstrated powerful at discriminating pathogenic from benign variants. However, its reliance on experimental structures and homologs limited its coverage of the proteome. We have now utilized AlphaFold2 models to develop MTR3D‐AF2, which covers 89.31% of proteins and 85.39% of residues across the human proteome. This work has improved MTR3D's ability to distinguish clinically established pathogenic from benign variants. MTR3D‐AF2 is freely available as an interactive web server at https://biosig.lab.uq.edu.au/mtr3daf2/.

## 1 BACKGROUND

Next generation sequencing technology has opened up new avenues for understanding disease, and subsequent precision medicine strategies. However, the effective use of this technology requires the ability to predict the pathogenicity of mutations with high accuracy. The typical human genome contains millions of single nucleotide variants (SNVs), making it a challenging task to distinguish the few pathogenic variants from the millions of benign within a patient (Consortium GP, 2015). The development of a computational tool to perform this task with high resolution is a key step towards the widespread implementation of precision medicine.

Researchers have approached this task primarily through developing computational tools which rely mainly on sequence conservation. Unfortunately, this approach still lacks sufficient robustness to be reliable in the clinic. Tools such as SIFT (Ng and Henikoff, 2003), PolyPhen2 (Adzhubei et al., 2013) and MutationTaster2 (Schwarz et al., 2014) provide readily available and easily accessible outputs which are used in a clinical setting (Cubuk et al., 2021) but are only regarded as weak evidence when multiple tools align in their prediction (Ernst et al., 2018; Richards et al., 2015). These computational tools have high sensitivity but low specificity. This makes them effective detectors of benign mutations, but not at competently identifying the few pathogenic variants among the millions of benign, making them ineffective clinical pathogenicity predictors (Cubuk et al., 2021). The recently released EVE (Frazer et al., 2021), ESM1b (Brandes et al., 2023) and AlphaMissense (Cheng et al., 2023) have a significantly improved accuracy over previous tools. However, given their novelty, their generalizability is yet to be tested for diagnostic reliability in the clinic.

Additionally, the majority of known variants still have an unknown clinical significance. Within the ClinVar database, this category currently amounts to approximately 52% of all variants (Henrie et al., 2018), highlighting the need for more informative approaches. Considering the costly and time consuming nature of experimentally assessing variants, the improvement and development of computational tools to perform pathogenicity predictions remains a promising path to apply population‐level data in diagnostics.

To address this, the missense tolerance ratio (MTR) was developed (Traynelis et al., 2017). Through the utilization of large datasets of human variation obtained from next generation sequencing, the ratio between the observed quantity of missense variation in the population and the expected amount at each residue is calculated. This score measures the degree to which residues are tolerant to missense mutations by comparing them to synonymous mutation enrichment in the same locus. The regions which are depleted of missense mutations in the population are so due to purifying natural selection, enabling the inference of biological importance of each residue. Residues with scores below 1 indicate that they have purifying selection acting upon them whereas those with scores above 1 indicate sites where variation has been selected for. A score of 1 indicates that the residue has the level of variation expected in the population considering only the mutation rate at that site.

Traditional measures of sequence conservation rely on a similar inference to MTR, however, these scores are calculated across species and measure the rate at which sequences are conserved across time. When used to assess the pathogenicity of human variants, MTR scores are advantageous over traditional measures of conservation since they are calculated using large datasets specific to the modern human population. MTR scores were originally calculated using amino acid sequence information (MTRv1 and MTRv2) (Silk et al., 2019; Traynelis et al., 2017) but the methodology was later modified to harness spatial information (MTR3D) which had been found to be an effective tool for gaining insights into the impact of point mutations (David & Sternberg, 2015; Nishi et al., 2016; Sahni et al., 2015; Stefl et al., 2013). Additionally, structural information had been successfully implemented in the prediction of pathogenicity (Iqbal et al., 2020). MTR3D's methodology relied on mapping genomic population data to the experimental structures found in the Protein Data Bank (PDB) (Berman et al., 2000) as well as homology based models (Silk et al., 2021). In both applications, regions with low MTR scores have been found to be significantly enriched in pathogenic variants, providing evidence of its utility in pathogenicity prediction. The integration of the MTR scores, along with structural features, into a machine‐learning model also produced an effective predictor of pathogenicity. This shows that mapping these scores onto their three‐dimensional protein environments offers a more informed representation of missense tolerance, however, it is also heavily reliant on an accurate protein structure. Currently, MTR3D, which utilized RCSB experimental crystal structures and those which could be modeled using SWISSMODEL (Schwede et al., 2003), only covers 60.85% of the residues in the human proteome, hindering widespread application of this metric.

Computationally predicting the three‐dimensional structure of proteins with experimental level accuracy was an aspiration for many years. In 2020, during the CASP14 (Kryshtafovych et al., 2021), AlphaFold2 (Jumper et al., 2021) demonstrated this ability. AlphaFold2's all atom accuracy, taking into account protein backbone and side chains, was 1.5 Å r.m.s.d.95 (95% confidence interval = 1.2–1.6 Å) while the best alternative method only achieved a 3.5 Å r.m.s.d.95 (95% confidence interval = 3.1–4.2 Å) (Jumper et al., 2021). Utilizing the outputs of this technology, we have substantially expanded the initial coverage of MTR3D across the human proteome and improved its performance in differentiating pathogenic from benign variants.

## 2 RESULTS

### 2.1 Coverage

**FIGURE 1:**
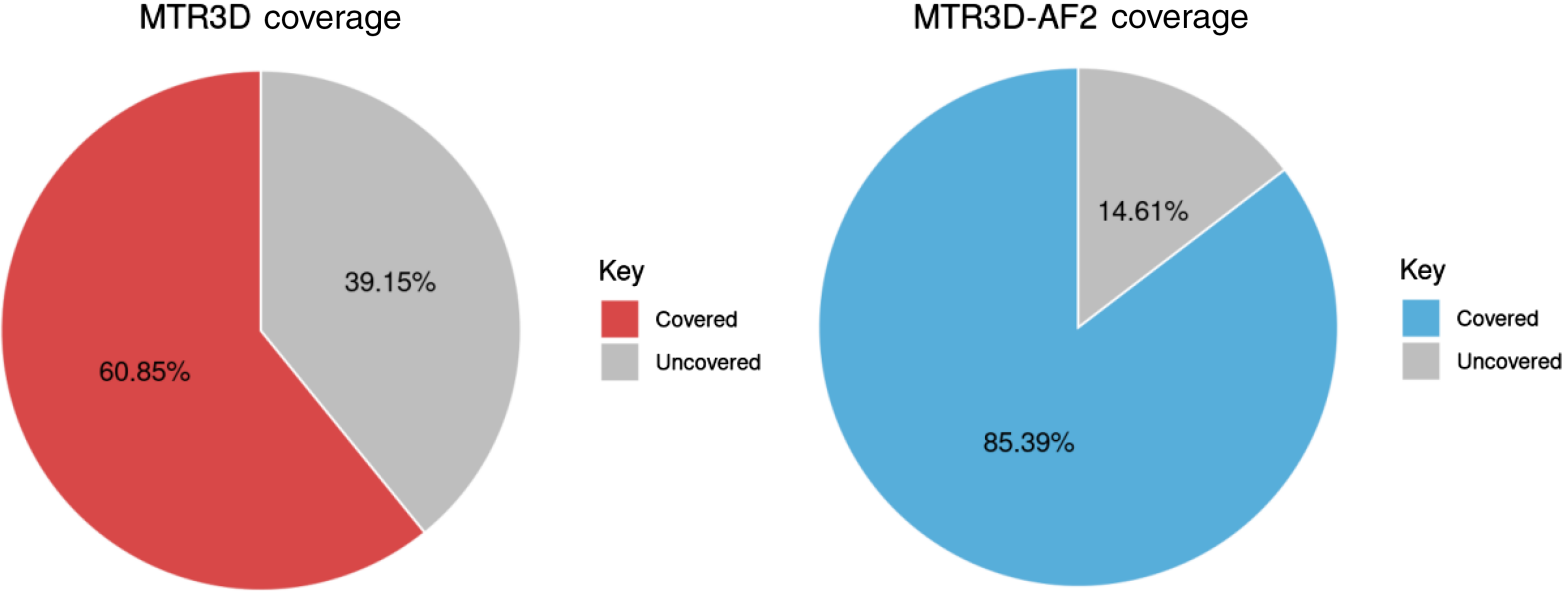
Pie charts showing the percentage of residues covered by (a) the original (MTR3D) and (b) updated versions (MTR3D‐AF2) of MTR both calculated using a window of 5 Å.

The human reference proteome harbors 20,518 unique proteins and 11,402,794 residues. The original MTR3D calculated using a 5 Å window covered 17,033 unique proteins (83.02%) and a total of 6,938,668 residues (60.85%). MTR3D‐AF2 calculated using a 5 Å window has an improved coverage of 18,324 (89.31%) unique proteins and 9,737,079 residues (85.39%) (Figure 1).

This large increase in residue coverage is due to the incomplete modeling of homology based and experimental structures present in MTR3D, in contrast to the full proteins modeled using AF2. Since the number of residues incorporated into each calculation is dependent upon the size of the window, the larger the window size, the greater the number of variation counts which are used to calculate the MTR score. For this reason, the MTR3D‐AF2 scores calculated using larger windows had fewer residues which lacked the sufficient variant counts to have a valid MTR3D‐AF2 score. The coverage of MTR3D‐AF2 calculated using a 14 Å window covered 9,869,961 residues (86.56%). The lack of coverage for the remaining 2174 proteins is due to the absence of GENCODE transcripts or complete AF2 models available for these proteins.

### 2.2 Validation

#### 2.2.1 *Mann–Whitney* U *tests*

The original MTR3D and the updated MTR3D‐AF2 scores which were aligned to the ClinVar set were used to perform a series of comparison and validation assessments. Specifically, a number of Mann–Whitney *U* tests were performed to determine if statistically significant differences in size exist between scores associated with pathogenic and benign variants. Statistically significant results, all with a *p*‐value <2.2e^−16^, were found when using every tested window size (5, 8, 11, and 14 Å). The comparison between scores associated with de novo pathogenic variants and non‐de novo pathogenic variants yielded the same results (*p*‐value <2.2e^−16^).

#### 2.2.2 MTR3D‐AF2 performance versus MTR3D

To determine the utility of MTR3D‐AF2 in discriminating pathogenic from benign variants compared to our original MTR3D we first compared the scores calculated using the 5 Å window. This comparison was performed to include only those sites which were covered by both versions. Considering the original MTR3D scores were calculated using all of the PDB structures and homology models available, in many cases there are multiple scores for the same residue in a protein. To enable a one‐to‐one comparison with MTR3D‐AF2, a mean MTR3D score was calculated at each site.

MTR3D calculated using a window of 5 Å had a mean score of 0.845 with a standard deviation of 0.212. The mean MTR3D‐AF2 score calculated using a window of 5 Å was 0.858 with a standard deviation of 0.260. To assess the difference in capability of MTR3D‐AF2 and MTR3D to differentiate pathogenic from benign variants a two‐proportion *z*‐test comparing the proportion of benign and pathogenic ClinVar variants in regions of intolerance (associated with a score of <0.5) was performed. After narrowing to only variants with at least two star quality, 41,974 benign variants and 24,686 pathogenic variants could be mapped to both MTR3D‐AF2 and MTR3D scores. These tests yielded *p*‐values of <2.2e^−16^ for each version of MTR and each window size. Since these results were incapable of separating the performance of the scores, the ratio of the percentage of pathogenic to the percentage of benign variants found in regions of intolerance was compared (Table 1).

**TABLE 1:** Table displaying the improvement in ratio of enriched pathogenic to benign ClinVar variants for differing window sizes using only those variants which had scores available for both versions.

	Benign intolerant	Benign total	Benign percentage	Pathogenic intolerant	Pathogenic total	Pathogenic percentage	Enrichment (fold change)	AUC
MTR3D‐AF2 5 Å	237	11,232	2.11	1166	6791	17.17	8.14	0.642
MTR3D 5 Å	74	6798	1.09	792	5992	12.17	11.18	0.662
MTR3D‐AF2 8 Å	112	11,226	1.00	941	6831	13.78	13.81	0.674
MTR3D‐8 Å	58	6950	0.84	633	6345	9.98	11.96	0.648

*Note*: The columns of this table includes (from left to right) the number of benign/pathogenic ClinVar variants of at least 2 star quality found in regions of intolerance for that window, the total number of benign/pathogenic ClinVar variants aligned to MTR3D‐AF2 scores, the percentage of benign/pathogenic ClinVar variants found in regions of intolerance, the ratio of the percentages of pathogenic to benign ClinVar variants found in regions of intolerance and finally the AUC calculated using the scores respective scores to predict the pathogenicity of the ClinVar variants.

When using the ratio of pathogenic to benign variants in regions of intolerance MTR3D‐AF2's performance was found to be worse (3.04 less). However, MTR3D‐AF2 scores calculated using the 8 Å had an improved performance in comparison to both the 5 Å (2.63 greater) and 8 Å window (1.85 greater). The AUC of MTR3D and MTR3D‐AF2 was calculated using the same set of ClinVar variants (Table 1). MTR3D had an AUC of 0.642 and 0.648 when calculated using a 5 Å window and 8 Å window respectively. MTR3D‐AF2 however, had an improved AUC of 0.662 and 0.674 when calculated using a 5 Å and an 8 Å window.

**FIGURE 2:**
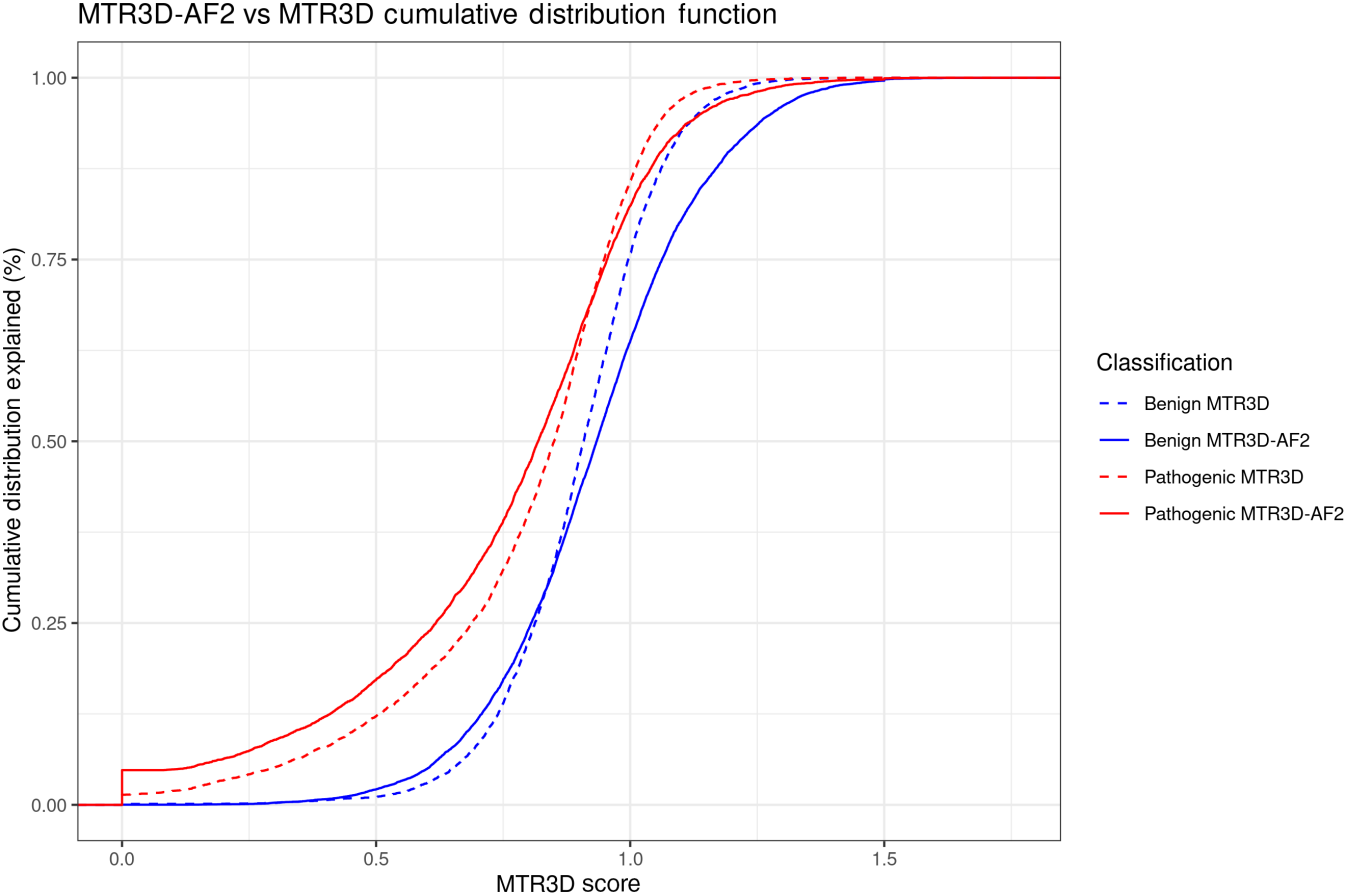
Cumulative distribution function plot displaying the distribution of MTR3D‐AF2 and MTR3D (original) scores calculated using a window of 5 Å associated with pathogenic and benign ClinVar variants.

**FIGURE 3:**
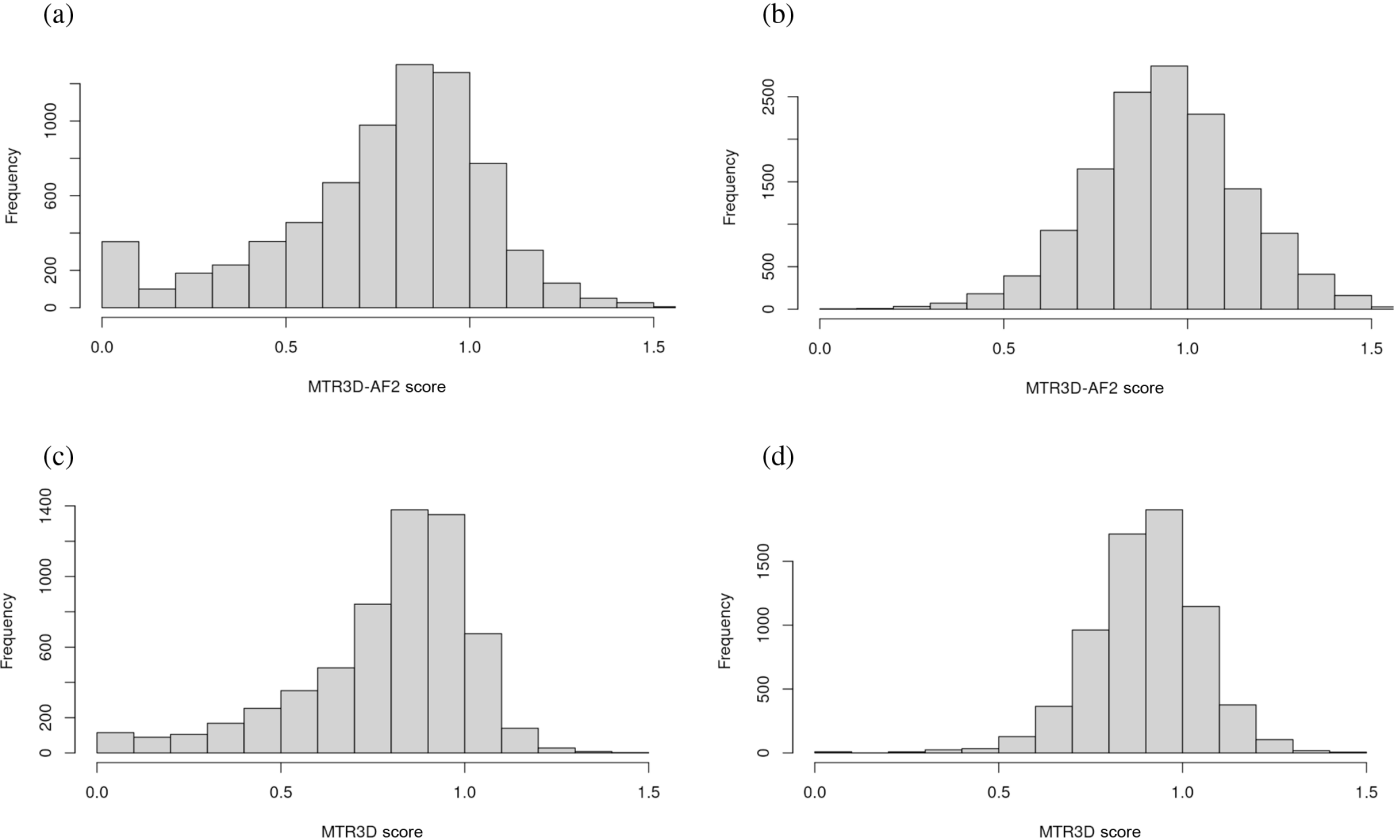
Histograms displaying distribution of scores associated with ClinVar variants. (a) MTR3D‐AF2 scores associated with pathogenic variants. (b) MTR3D‐AF2 scores associated with benign variants. (c) MTR3D scores associated with pathogenic variants. (d) MTR3D scores associated with benign variants.

This improvement in discriminating between pathogenic and benign variants is further demonstrated in the distributions of both MTR scores calculated using a 5 Å window associated with benign and pathogenic variants. There is an observably consistent separation in the cumulative distribution plot (Figure 2) between the pathogenic MTR3D‐AF2 (solid red) and benign MTR3D‐AF2 (solid blue) lines compared to their MTR3D (dashed) counterparts throughout the entire distribution. When observing the distributions in histogram form (Figure 3), it is clear that the difference is greatest at the ends of the distributions. Many pathogenic variants have MTR3D‐AF2 scores below 0.1 (most of these are 0) while very few have MTR3D scores below 0.1. There is a greater number of MTR3D‐AF2 scores associated with pathogenic and benign variants above 1 compared to MTR3D scores.

These observations can be explained by considering the expanded coverage of disordered regions, which are typically situated at the periphery of the protein and often predicted to be linearly extending away from the rest of the protein. This phenomena means that residues in disordered regions often have fewer residues captured in their windows, leading to less data utilized in their calculations. This, in turn, gives rise to a greater number of scores in the extreme ends of the distribution.

#### 2.2.3 MTR3D‐AF2 performance versus MTR

An analysis was also performed to compare the amount of pathogenic variants found within regions of intolerance compared to benign for both MTR3D‐AF2 calculated using an 8 Å window and MTRv2 (sequence‐based) using a 31 amino acid window. When including only those ClinVar variants covered by both scores and curated to only variants with at least a two star quality there were 16,329 benign and 8524 pathogenic variants. MTRv2 was found to be able to discriminate pathogenic variants from benign in these areas at a rate far superior to MTR3D‐AF2. Using MTR3D‐AF2, the ratio of the percentage of pathogenic variants in regions of intolerance compared to benign in these regions was greater by 12.227 compared to the ratio of 42.547 when using MTRv2.

**FIGURE 4:**
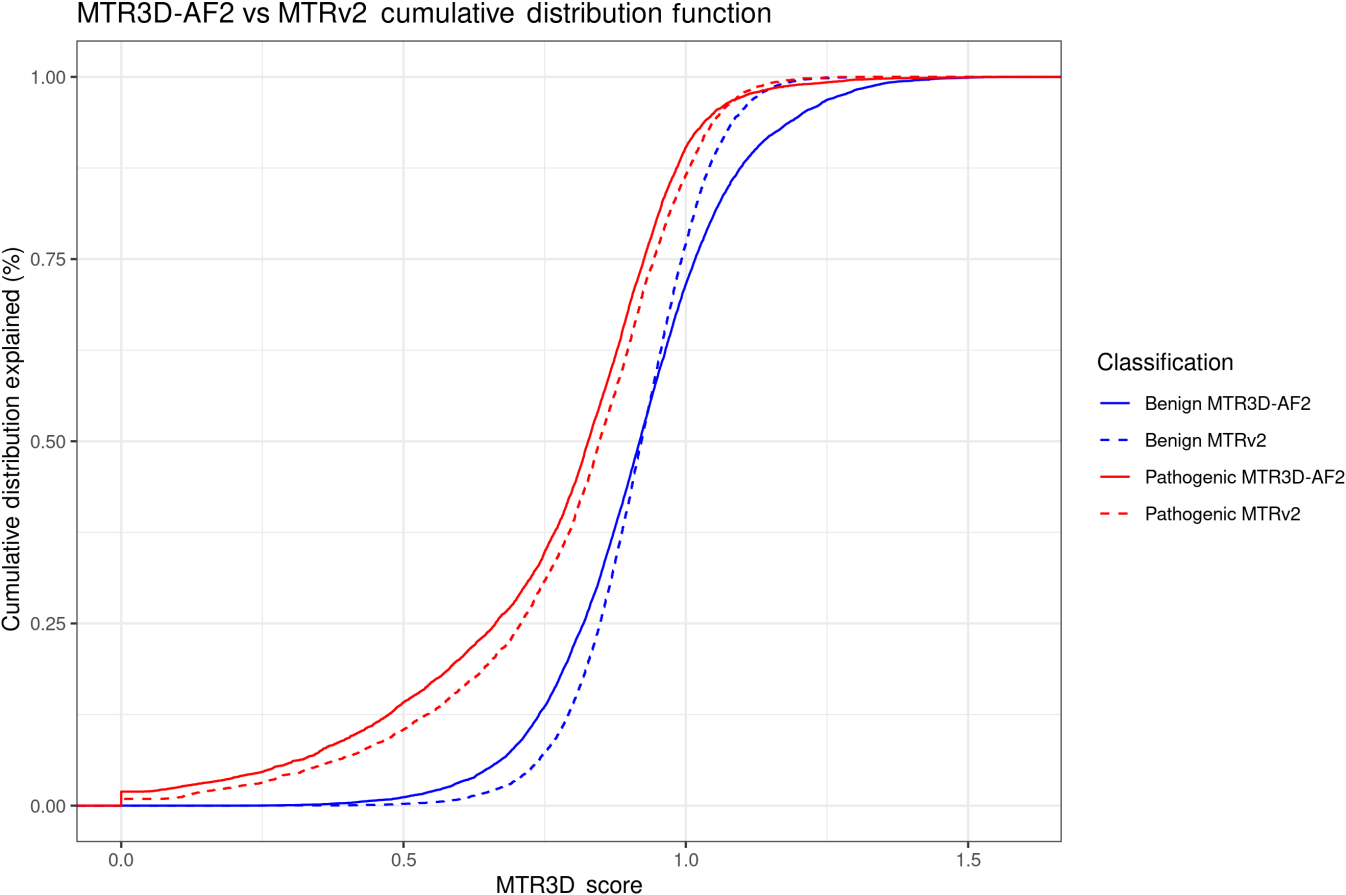
Cumulative distribution function plot displaying the distribution of MTR3D‐AF2 and MTRv2 (sequence‐based) scores calculated using a window of 8 Å associated with pathogenic and benign variants.

When examining the cumulative distribution plot comparing MTR3D‐AF2 and MTRv2 (Figure 4), however, it is observable that there is a greater degree of separation between the MTR3D‐AF2 pathogenic (solid red) and benign (solid blue) lines compared to their MTRv2 (dashed) counterparts in regions above 0.75. To measure the degree to which the two scores can be used to make predictions of pathogenicity, the AUC for each was calculated. MTR3D‐AF2 calculated using a window of 8 Å on this set of ClinVar variants was found to have an AUC of 0.684 compared to MTRv2's AUC of 0.666.

#### 2.2.4 MTR3D‐AF2 performance versus COSMIS

Next a comparison with COSMIS (Li et al., 2022), a constraint based score developed in 2022, which leverages gnomAD v2.1.1 and is calculated using a similar method to MTR, was performed. We have obtained the same set of ClinVar variants which COSMIS used for comparisons with other constraint‐based scores from the supplementary data. The variant set was then filtered to include only those which were covered by both MTR3D‐AF2 and COSMIS. When calculating AUC on this set of variants, the 5, 8, 11, and 14 Å were tested and the 14 Å window was found to perform the best, with an AUC of 0.701 compared to COSMIS’ AUC of 0.730.

COSMIS uses experimental structures from the PDB as well as models from the SWISS‐MODEL repository (Bienert et al., 2017) to provide structural information and only relies on AlphaFold2 models when neither of these are present. We hypothesized that the ability to rely on scores from a combination of experimental, homology based and AlphaFold2 models to provide structural information gives an advantage to MTR3D. To test this, for each protein the accuracy of MTR3D and MTR3D‐AF2 was calculated. The score for each protein which had the highest accuracy was included in the set of scores used to calculate an overall AUC. Using this methodology, MTR3D and MTR3D‐AF2 together produced an AUC of 0.732. Performing the analysis this way reduced the number of variants which could be tested from 35,559 to 35,158, due to limited coverage of MTR3D. COSMIS had an AUC of 0.736 on this set of variants.

#### 2.2.5 Comparison of windows

To determine the degree to which the different window sizes are capable of discriminating between pathogenic and benign variants, scores were calculated using window sizes 5, 8, 11, and 14 Å (Table 2). To assess the discriminatory power of these differing window sizes we compared the enrichment of pathogenic and benign missense ClinVar variants in regions of intolerance using again the ratio of the percentages.

**TABLE 2:** Comparison of enrichment of pathogenic versus benign variants for differing window sizes.

	Benign intolerant	Benign total	Percentage	Pathogenic intolerant	Pathogenic total	Percentage	Enrichment (fold change)	AUC
MTR3D‐AF 5 Å	287	13,886	2.07	1220	7186	16.98	8.21	0.698
MTR3D‐AF 8 Å	152	13,903	1.09	981	7218	13.59	12.43	0.690
MTR3D‐AF 11 Å	80	13,906	0.58	808	7219	11.19	19.46	0.700
MTR3D‐AF 14 Å	64	13,908	0.46	656	7220	9.09	19.75	0.699

*Note*: The columns of this table includes (from left to right) the number of benign/pathogenic ClinVar variants of at least 2 star quality found in regions of intolerance for that window, the total number of benign/pathogenic ClinVar variants aligned to MTR3D‐AF2 scores, the percentage of benign/pathogenic ClinVar variants found in regions of intolerance, the ratio of the percentages of pathogenic to benign ClinVar variants found in regions of intolerance and finally the AUC calculated using the scores respective scores to predict the pathogenicity of the ClinVar variants.

Performing a two‐proportion *z*‐test comparing the proportion of benign and pathogenic variants in regions of intolerance again yielded *p*‐values of <2.2e^−16^ for each window size. The ratio of pathogenic variants to benign variants found in regions of intolerance was found to be improved with diminishing returns by increasing the size of the window. The improvements were greatest when increasing the window size from 5 Å (8.21) to 8 Å (12.43) and then from 8 to 11 Å (19.46). Increasing the window size up to 14 Å (19.75) only improved the degree of discrimination marginally. To compare the scores further, the AUC was calculated for each window size (Table 2). All scores were found to be very similar in their use as a pathogenicity predictor, however the 11 Å window (0.700) had the highest performance, only 0.01 AUC higher than the 14 Å window (0.6).

**FIGURE 5:**
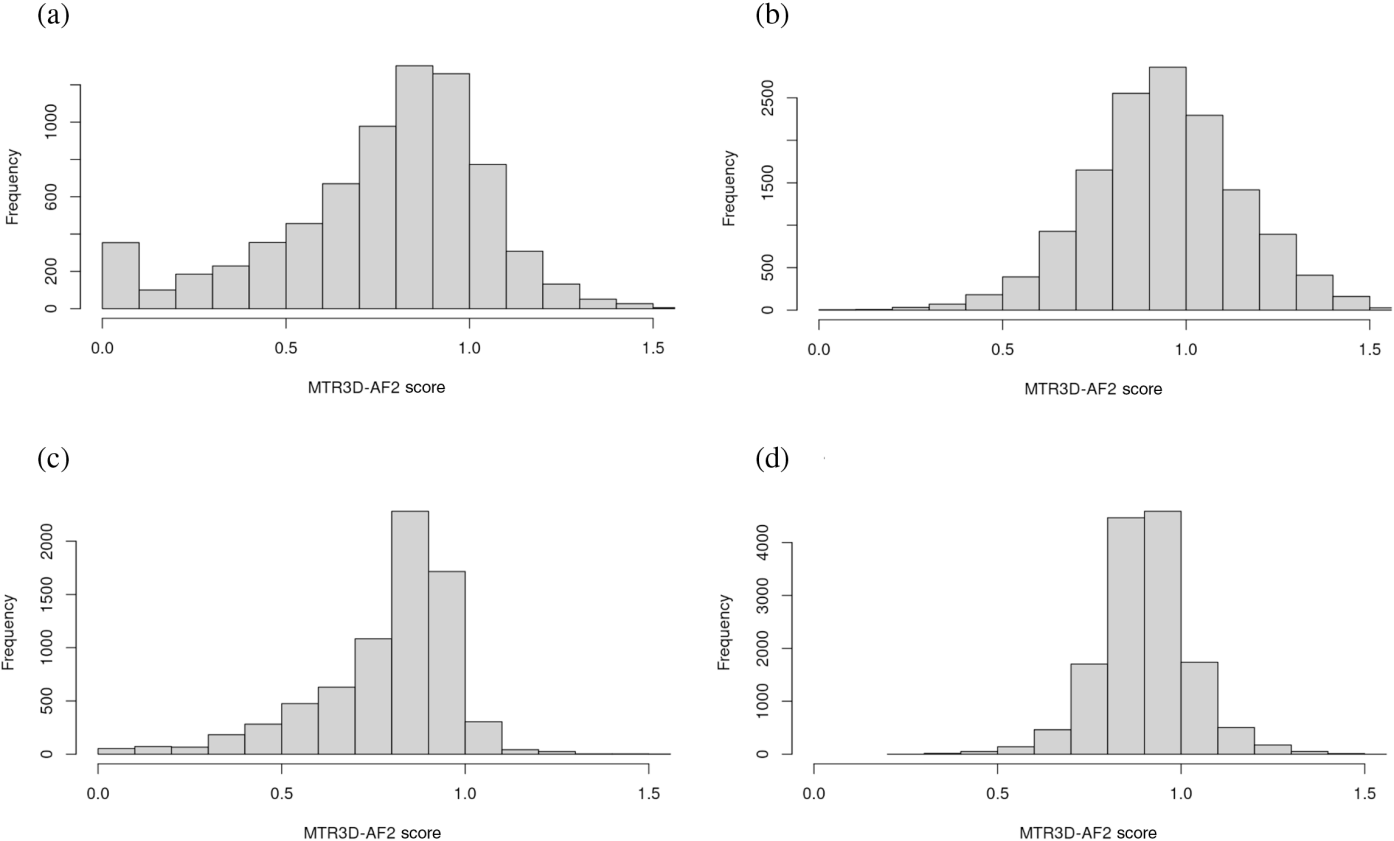
Histograms displaying the distribution of scores associated with pathogenic and benign variants calculated using two window sizes. (a) Scores calculated using a 5 Å window associated with pathogenic variants. (b) Scores calculated using a 5 Å window associated with benign variants. (c) Scores calculated using a 14 Å window associated with pathogenic variants. (d) Scores calculated using a 14 Å window associated with benign variants.

As the size of the window grew, the distribution of scores associated with both pathogenic and benign variants shifted both ends of the distributions closer to the center of the curve. When comparing the 5 Å score distribution to the 14 Å distribution (Figure 5), this shift results in most of the benign variants moving out of regions of intolerance, leaving less than 25% of the variants found in these regions when using the 5 Å window. While the shift also reduces the percentage of pathogenic variants found in regions of intolerance, the reduction is less than 50%, resulting in a greater number of pathogenic variants found in this region of intolerance. We can also observe that this shift results in there being very few pathogenic variants associated with scores above 1 calculated using the 14 Å window (Figure 5).

**FIGURE 6:**
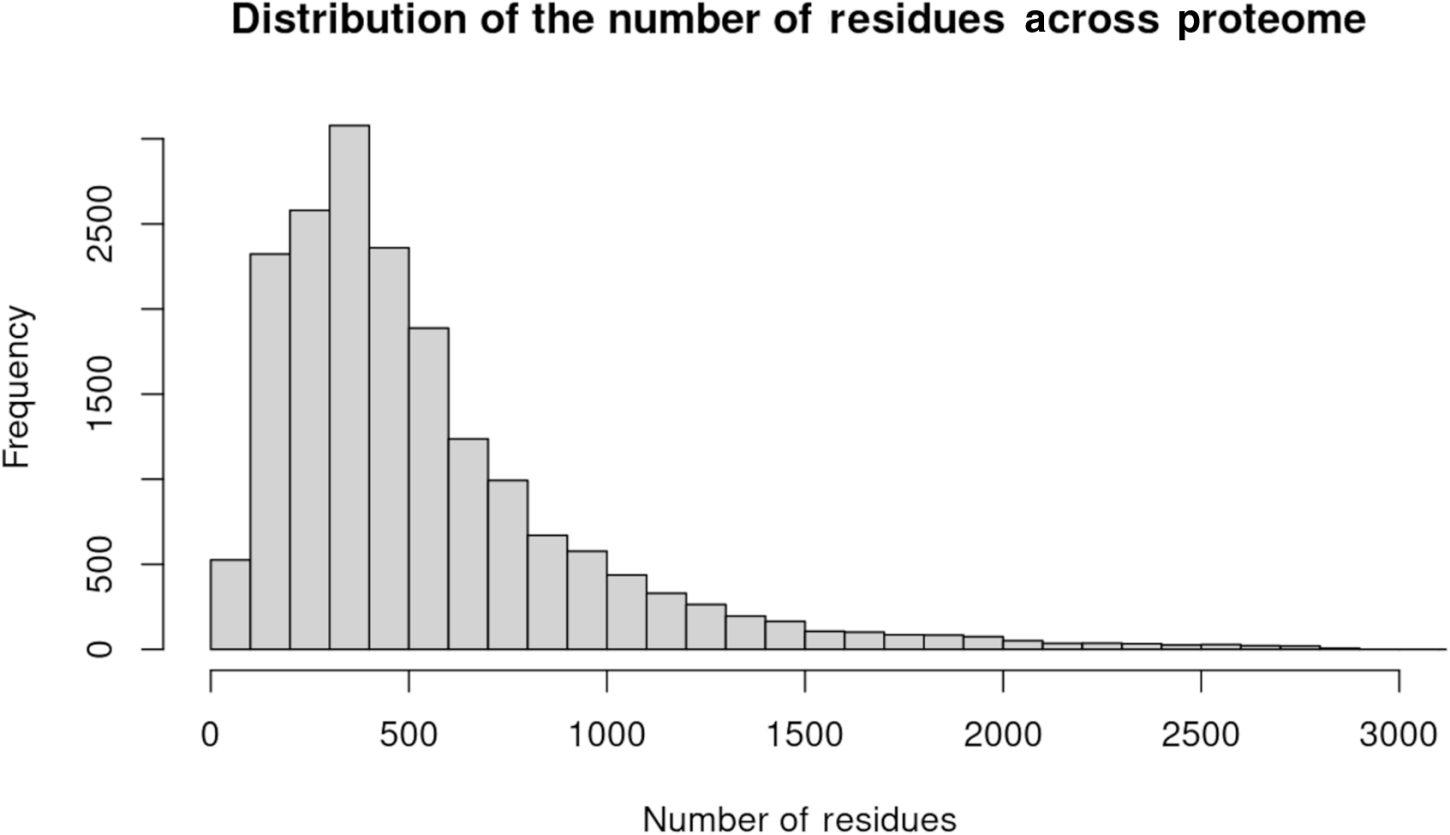
Histogram displaying the distribution of the size of proteins in number of residues across the human proteome.

**FIGURE 7:**
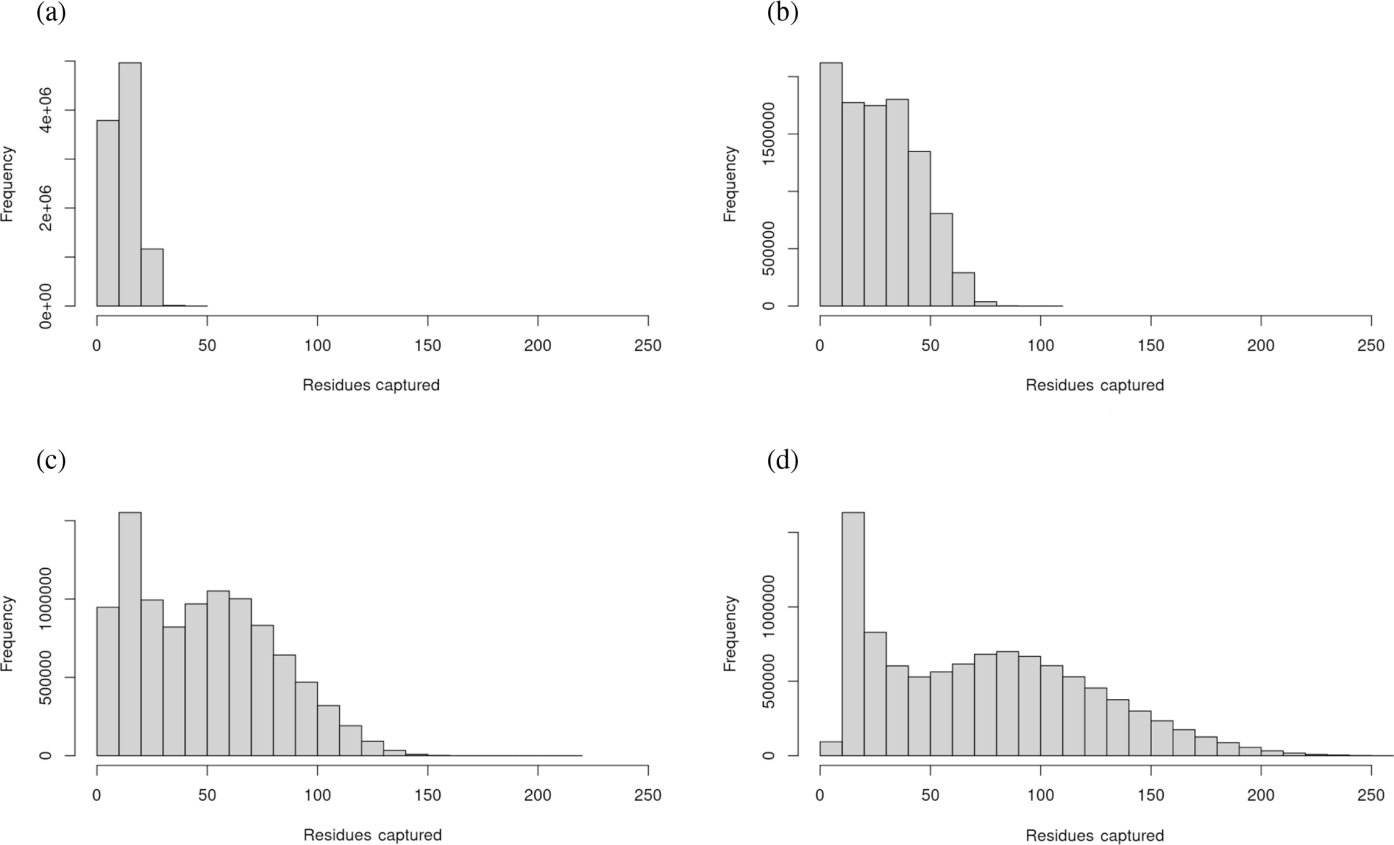
Histogram of the distribution of sizes of proteins in residues across the human proteome. (a) Residues captured by 5 Å window, (b) residues captured by 8 Å window, (c) residues captured by 11 Å window, and (d) residues captured by 5 Å window.

To better understand why larger size windows might be performing better at discriminating pathogenic from benign variants in regions of intolerance, the mean number of residues captured within the windows for each residue were calculated for the 5, 8, 11, and 14 Å windows. The mean numbers captured were 12.74, 28.24, 49.23, and 74.79, while the median numbers were 12, 27, 47, and 72 respectively. To put this size in context, the mean and median number of residues in a protein across the human proteome is 542 and 426, respectively. Histograms were also created to visualize the distribution of residues captured by windows across the whole proteome (Figure 6) as well as a histogram representing the distribution of the number of residues in each protein in the proteome (Figure 7). These figures illustrate the broad range of protein sizes and number of residues captured using larger window sizes.

The mean number of residues captured by the 5 Å window offers an explanation of its underperformance in discriminating between pathogenic and benign variants. Within the sequence‐based version, the larger 41 amino acid window was a better predictor of pathogenicity when compared to the 21 amino acid window (Silk et al., 2021). This therefore suggests that window sizes capturing only 12 residues on average (5 Å) would lack the variant counts necessary to perform the calculation with sufficient power.

#### 2.2.6 Performance in de novo variants

MTR3D had a superior performance in differentiating de novo pathogenic variants from benign variants compared to non‐de novo pathogenic variants. When assessing MTR3D‐AF2 scores at 8 Å, our Mann–Whitney *U* test reconfirmed this capability in discerning between de novo and non‐de novo pathogenic variants (*p*‐value: 2.2e^−16^).

**FIGURE 8:**
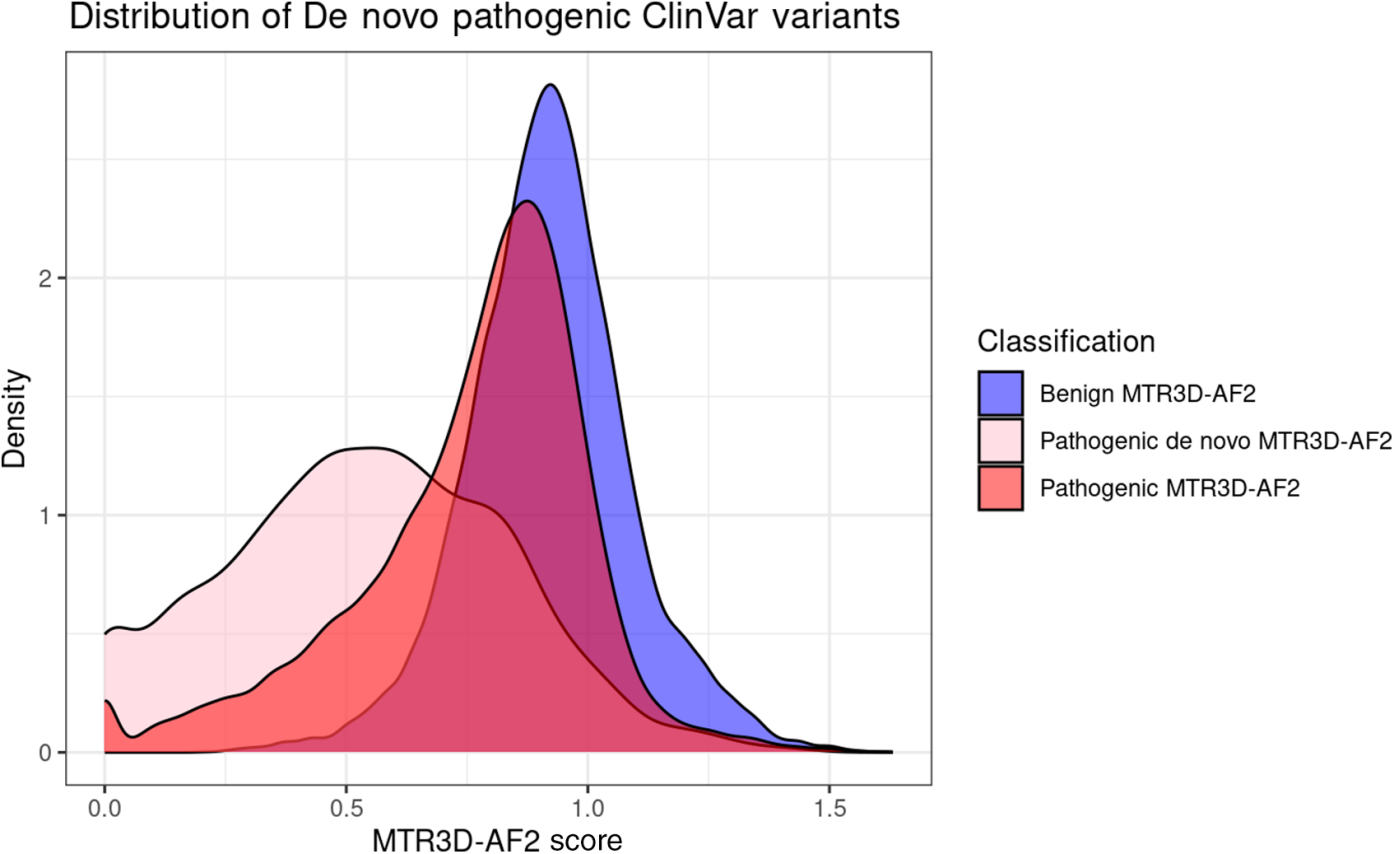
Overlapping histogram of MTR3D‐AF2 scores calculated using 8 Å window associated with benign variants (blue), pathogenic de novo variants (pink) and non‐de novo pathogenic variants (red).

Further supporting evidence can be observed in the mean MTR3D‐AF2 scores, calculated using an 8 Å window. For non‐de novo variants, the mean score was 0.742, while de novo variants exhibited a notably lower mean score of 0.484. Additionally, the overlapping distributions of de novo pathogenic variants, non‐de novo pathogenic variants and benign variants (Figure 8) shows the large degree of separation between de novo variants and the other two distributions.

#### 2.2.7 Low pLDDT regions

After applying three filters to differentiate scores calculated using a window of 14 Å to those associated with high, medium, and low quality structure predictions, the corresponding Mann–Whitney *U* tests comparing scores associated with pathogenic and benign variants had *p*‐values of 2.2e^−16^ (*n =* 13,398), 2.2e^−16^ (*n =* 1770), and 4.205e^−13^ (*n* = 5949), respectively. While all of these results are significant, there is a reduced p‐value in those MTR3D‐AF2 scores found in regions with low predicted local distance difference test (pLDDT) scores. To assess the relationship between MTR3D scores calculated using an 8 Å window and pLDDT scores a correlation coefficient was calculated at −0.145 using the Pearson method. The inverse relationship between MTR3D‐AF2 and pLDDT scores aligns with the expectation that functionally important sites are likely to be found in more highly structured regions, although exceptions have been reported (Vacic et al., 2012).

**FIGURE 9:**
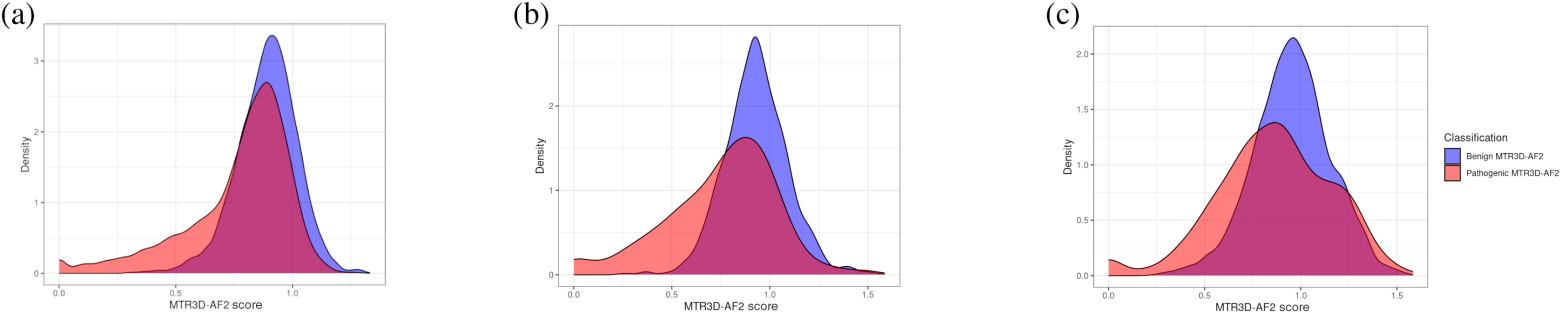
Overlapping distributions of MTR3D‐AF2 scores calculated using an 8 Å window associated with pathogenic and benign variants (a) filtered to only those variants found in regions with pLDDT scores of >0.7, (b) filtered to only those variants found in regions with pLDDT scores between <0.7 and >0.5, and (c) filtered to only those variants found in regions with pLDDT scores of <0.5.

The overlapping distributions of scores associated with benign and pathogenic variants after filtering for different pLDDT scores provides further insight into the reliability of the MTR3D‐AF2 scores found in low confidence regions (Figure 9). While the medium and high quality distributions show a similar level of overlap between the pathogenic and benign associated scores, the low quality sites are largely overlapping. Of additional note is the difference between the distributions of pathogenic and benign variants associated with MTR3D‐AF2 scores above 1 in the high quality pLDDT score distribution compared to the medium and low quality filtered distributions. There is a progressively increasing number of both pathogenic and benign variants associated with MTR3D‐AF2 scores above 1 with the decreasing of quality prediction filters. This is likely due to lower quality structural predictions lying on the outer edges of structures meaning that fewer variant counts are included in their calculation resulting in more extreme scores.

### 2.3 Web server

We have implemented MTR3D‐AF2 as a user‐friendly and freely available web‐server (https://biosig.lab.uq.edu.au/mtr3daf2/). The server front end was developed via Materialise CSS framework version 1.0.0, and the back end was built in Python 3.6 via the Flask framework (version 0.12.3). It is hosted on a Linux server running Nginx (version 1.23.0).

#### 2.3.1 Input

Users can search for their protein of interest using either the UniProt Accession code or the UniProt ID. Alternatively, the user can search for their Ensembl transcript of interest via the same search bar. The user may also access the full database of MTR3D‐AF2 and MTRv2 scores that are available as a bulk download in the datasets section.

#### 2.3.2 Output

An interactive line graph generated with bokeh is displayed which shows the scores of the current MTR version selected, these being either MTR3D‐AF2 or MTRv2, across the residues of the protein of interest. Below this is a viewer built to interact with the protein structure and visualize the scores mapped to it. The MTR score is scaled from red, indicating intolerance, to blue, indicating tolerance. Regions in white indicate the approximate average level of tolerance across the proteome, that being mildly intolerant to missense mutation (MTR score of 0.85). Using the drop down arrow, those interested can also visualize the pLDDT scores mapped to the structure to gain an idea of the AF2 confidence in its prediction across the model.

The user can search for and select a specific residue of the protein to be highlighted with a red dot on the line graph and displayed in stick format on the structure view. The MTR scores and structure for the protein of interest can be downloaded at the bottom of the page. A screenshot of the model can also be taken in the position which the user orients and highlights it.

In the bulk downloads section there is a downloadable file which contains all available scores for each residue site calculated using each window size along with which amino acid is found here. There are also 4 additional files which have the MTR3D‐AF2 scores aligned to each of the ClinVar variants which could be mapped, one file for each of the window sizes (5, 8, 11, and 14 Å).

## 3 DISCUSSION

The application of our previously derived spatial score, MTR3D, to the whole proteome using AlphaFold2 derived structures successfully resulted in an improvement in score coverage. While an increase of 1291 proteins covered is not insignificant, the more substantial improvement lies in the calculation of scores for proteins which were previously partly unmodeled experimentally or modeled using close homologs. Considering that the score accounts for variation patterns across neighboring residues, the impact of missing structural regions extends beyond the region itself.

Interestingly, we observed a notably higher ratio of pathogenic to benign variants which could be mapped to MTR3D scores compared to those mapped to MTR3D‐AF2. The explanation for this is that because MTR3D relies primarily on experimental structures, these scores cover a limited proportion of disordered regions, where benign variants are more likely to lie. While a substantial (21.7%) (Vacic et al., 2012) amount of pathogenic variants are still found within disordered regions, in considering whole protein models through AF2, previously disordered and predominantly benign‐enriched regions became available for calculation, shifting this proportion.

We have also noticed that the distribution of the number of residues captured by the three‐dimensional window explains the degree to which the residue structural location, along with the local fold, can alter the number of variant counts involved in the calculation for each residue. This means that, while in the sequence‐based version of MTR there is consistency in the number of residues captured, this is not the case for the spatial versions of MTR. Despite the 14 Å window being very large and often capturing more than 100 amino acids, there are a large number of residues where 14 Å windows capture less than 50 residues. Importantly, apart from the 11 Å windows, there are very few sites with windows which capture less than 10 residues. While using spatial information offers a better representation of the residue environment when compared to the sequence‐based approach, the variability in residues captured across proteins at the same window size means that for many residues their calculations may lack sufficient variation counts. This may explain why the larger window sizes display an improved capability to discriminate between pathogenic and benign variants.

Considering that both the 11 and 14 Å window are quite large, their improved discriminatory power may come from more general tolerance information about the gene rather than information local to the residue in question. However, as the 11 Å window provides similar discrimination between pathogenic and benign variants to the 14 Å window, the identification of an appropriate window size, in practice, would depend on the score utility. Calculating the AUCs for each of the window sizes provides evidence that shifting the window sizes may not alter the performance of the scores substantially. While a machine‐learning test performed by training different models on various window sizes and evaluating the pathogenicity prediction of the models will likely provide superior elucidation on the ideal window size, this was deemed beyond the scope of this current work.

While all of the Mann–Whitney *U* tests yielded statistically significant results after filtering for different pLDDT scores, it is worth noting that such statistical tests which are performed on large datasets are sensitive to small differences. The increased overlap in distribution of scores associated with pathogenic and benign variants in low confidence prediction sites, in combination with the link between low quality pLDDT scores and MTR3D‐AF2 scores above 1 provides evidence of the unreliability of MTR3D‐AF2 scores associated with residues in these regions. While the scores of residues in disordered regions may give us some insight into missense tolerance, the implicit reliance on these scores would depend on the extent of their utility in practice. For more detailed assessments of mutational tolerance in these regions, we suggest a combinatorial approach using MTR3D‐AF2 and sequenced based scores as disordered regions tend to be more dynamic, making their behavior and impact on neighboring residues less easy to account for. For ease of use, we have also made our sequence‐based scores accessible through our web server, available for each transcript.

The improvement in discriminatory power of MTR3D‐AF2 compared to the original version of MTR3D is substantial and likely due to the difference in quality between the homology‐based models and the AlphaFold2 predicted models. For MTR3D, 23.1% of the homology models used for the calculation were considered poor quality, as measured using *Z*‐scores (where values less than −4 were considered low quality). Whereas, in MTR3D‐AF2, only 3.4% of AlphaFold2 models were considered low quality (mean pLDDT score <50). While the categorization of prediction quality is not assessed in the same way for each of these tools, these numbers provide a rough estimate in the improvement of model accuracy of AlphaFold2 over SWISS‐MODEL, and by extension, the improvement of MTR3D scores when applied to AlphaFold2 structures, described in this work.

It should be noted that both COSMIS and MTR3D‐AF2 are outperformed by ConSurf, a measure of conservation across species (Ashkenazy et al., 2016), by greater than 0.1 AUC. However, the authors of COSMIS demonstrated that integrating COSMIS, the previous versions of MTR, ConSurf and other predictive scores together into a machine learning model produced a predictive tool superior to any of the scores alone (Li et al., 2022). For this reason we believe MTR3D‐AF2's novel approach to the prediction of variant deleteriousness will allow it to serve as a useful addition to the current set of pathogenicity prediction tools.

## 4 CONCLUSIONS

MTR calculations leveraging spatial information have demonstrated their effectiveness in discriminating between pathogenic and benign variants. However, their utility was previously constrained to regions of the human proteome that had undergone experimental evaluation or shared close homologs. In the latest iteration, MTR3D‐AF2, we have incorporated AlphaFold2's near‐experimental level protein structure predictions to significantly enhance both coverage and predictive power. The integration of MTR3D‐AF2 into machine learning‐based models holds promise for further advancements in the field of pathogenicity prediction.

## 5 METHODS

### 5.1 Aim

The aim of this methodology was to address the deficiencies in MTR3D score coverage by leveraging AlphaFold2 models to perform their calculations.

### 5.2 Attainment of AlphaFold2 models

The AlphaFold2 pre‐modeled structures were downloaded from the AlphaFold website in October 2022 (Varadi et al., 2022). The AlphaFold2 team have modeled and made available all structures of the human proteome, however 208 of these were only modeled in segments due to protein size and ensuing computational constraints. To further enhance the coverage of the proteome, we successfully modeled the full structure of the smallest 27 proteins (2040–2813 AA) from the remaining 208 using AlphaFold2 version 2.2.4 taken from the GitHub repository. In doing so, the full_dbs option was used to search for suitable templates, which contained .pdb files available until the cut‐off date of 10‐09‐2022, while the remaining parameters were kept as default. Five models were constructed by AF2 and the structure with the highest pLDDT scores was used for all of the calculations. Each protein was modeled based on the canonical sequence, taken from the uniprot reference proteome UP000005640.

### 5.3 Missense tolerance ratio calculations

The MTR3D score was calculated for each residue of every protein. For every calculation, a variable size angstrom sphere was created with the residue in question at the center, representing the three‐dimensional environment window. Sphere sizes tested were 5, 8, 11, and 14 Å. The MTR3D calculation, which incorporates the missense and synonymous mutation counts for each of the surrounding residues captured within the window, is as follows:

(1) $$\left(\mathrm{MT}R_{i}=\frac{\text{missense}\_\mathrm{ob}s_{i}/\left(\text{missense}\_\mathrm{ob}s_{i}+\text{synonymous}\_\mathrm{ob}s_{i}\right)}{\text{missense}\_\mathrm{ex}p_{i}/\left(\text{missense}\_\mathrm{ex}p_{i}+\text{synonymous}\_\mathrm{ex}p_{i}\right)}\right)$$

Including synonymous observations into the calculation serves as a variation control, to account for the mutation rate of the sites measured. This calculation was performed for every available GENCODE (Frankish et al., 2021) transcript (*n* = 27,077) which had a corresponding AlphaFold2 structure modeled.

The FASTA sequences of each protein from the reference proteome were downloaded from the uniprot website and aligned to the GENCODE transcripts V43. Observed variant data was obtained from gnomAD v2.1.1 (Karczewski et al., 2020) (*n* = 125,748 exomes and *n* = 15,708 genomes), Biobank (Sudlow et al., 2015) (*n* = 200,000 exomes) and DiscovEHR (Dewey et al., 2016) (*n* = 50,000 exomes). As DiscovEHR mutant data was our only data source aligned to the GRCh37 genome, the LiftOver (Haeussler et al., 2019) software was used to convert this dataset to the GRCh38 genome, in line with our other data. The curated samples were next run through Variant Effect Predictor (VEP) v109 (McLaren et al., 2016) to enable variant annotation from the nucleotide‐ to protein‐level, where overall data was filtered to include missense and synonymous mutations, as required by the MTR calculation. These outputs were then mapped to the GENCODE transcripts which could then be mapped back to the AF2 structures. The expected variant data was simulated to contain every possible missense and synonymous mutation at each point in the aligned transcripts, and was subsequently annotated by VEP.

Residues which had fewer than five missense or synonymous observed mutations associated with all of the residues within their window combined were considered to have an insufficient amount of data to calculate a valid MTR3D‐AF2 score and were discarded. Within the 5 Å window, this represented 1.91%, which decreased with every increase in window size down to 0.58% in the 14 Å window.

### 5.4 MTR‐AF2 validation

To validate the MTR3D‐AF2 scores and compare them with the previous version of MTR3D, variants from ClinVar (Landrum et al., 2018) (accessed on March 29, 2023) were aligned to the positions in each of the transcripts which had MTR3D and MTR3D‐AF2 scores attached. These ClinVar variants were filtered to only those which were missense and had been submitted by multiple authors without conflicting interpretations, as to ensure only reliable variants were used in our analyses. The R packages ensembldb v2.18.4 (Rainer et al., 2019) and EnsDb.Hsapiens.v86 v2.99.0 were used to map the ClinVar variants from their genomic position to Ensembl transcripts. Mann–Whitney *U* tests were performed using R to determine if statistically significant differences existed between the set of MTR3D‐AF2 scores associated with pathogenic variants and those associated with the benign variants. Similarly, a Mann–Whitney *U* test was performed to see if there was a statistically significant difference between MTR3D‐AF2 scores associated with Pathogenic variants occurring de novo, versus those variants associated with non‐de novo pathogenic variants. To assess the ability of the two versions of MTR3D as well as the different window sizes to differentiate pathogenic from benign variants, a series of two‐proportion *z*‐tests comparing the proportion of benign and pathogenic variants in regions of intolerance was performed. The R package pROC was used to calculate the AUCs of all scores.

The AlphaFold team considers pLDDT scores more than 70 to be a generally good prediction of residue position in three‐dimensional space and those less than 50 to be a poor prediction (Jumper et al., 2021). We performed Mann–Whitney *U* tests to compare the scores calculated using an 8 Å window associated with pathogenic and benign variants after filtering for pLDDT scores more than 70 (high quality), less than 70 but more than 50 (medium quality) and less than 50 (poor quality) to determine the usefulness of MTR3D‐AF2 scores associated with residues that had varying pLDDT scores. All figures including the pie chart, the series of histograms and the cumulative distribution function plot were all created using R. All of the MTR calculations and the majority of the data processing were performed using R and the rest using bash.

## AUTHOR CONTRIBUTIONS

**Aaron Kovacs:** Methodology; software; formal analysis; validation; writing – original draft; visualization; data curation. **Stephanie Portelli:** Methodology; validation; writing – review and editing. **Michael Silk:** Methodology; writing – review and editing; data curation. **Carlos H. M. Rodrigues:** Visualization; software; writing – review and editing. **David B. Ascher:** Conceptualization; methodology; writing – review and editing; supervision.

## FUNDING INFORMATION

This research was funded by Investigator Grant from the National Health and Medical Research Council (NHMRC) of Australia [GNT1174405] and Victorian Government's Operational Infrastructure Support Program (in part).

## CONFLICT OF INTEREST STATEMENT

The authors declare that they have no competing interests.

## Data Availability

The MTR3D‐AF2 scores generated during the current study are available in the MTR3D‐AF2 repository, available at https://biosig.lab.uq.edu.au/mtr3daf2/. The gnomAD v2 dataset is available from the gnomAD website, https://gnomad.broadinstitute.org/downloads#v2‐liftover. The discovEHR dataset is no longer publicly available and may be available by contacting the corresponding author of the dataset. This research has been conducted using the UK Biobank Resource under Application Number 50000. Funding for open access charge: MRC.
